# Prognostic Value of a Novel Staging System Integrating Lymph Node Station Number and Tumor Regression Grade for Esophageal Cancer Following Neoadjuvant Chemoradiotherapy

**DOI:** 10.1245/s10434-025-18612-y

**Published:** 2025-11-09

**Authors:** Bo-Wei Liu, Wen-Dan-Qi Liang, Yi-Min Gu, Han-Lu Zhang, Qi-Xin Shang, Long-Qi Chen

**Affiliations:** 1 Department of Thoracic Surgery, West China Hospital of Sichuan University, Chengdu, China; 2 Laboratory of Anaesthesia and Critical Care Medicine, National-Local Joint Engineering Research Centre of Translational Medicine of Anaesthesiology, West China Hospital, Si Chuan university, Chengdu, China; 3 Department of Thoracic Surgery, West China Hospital of Sichuan University, Chengdu, China

**Keywords:** Neoadjuvant chemoradiotherapy, ESCC, Tumor regression grade

## Abstract

**Background:**

The aim of this study was to evaluate the prognostic value of tumor regression grade (TRG) in patients with esophageal cancer after neoadjuvant therapy and to validate the prognostic value of a novel TRG system (nTRG) system including the number of metastatic lymph node stations (S system) in patients with esophageal cancer after neoadjuvant therapy.

**Method:**

A total of 514 patients were enrolled in the study cohort, and survival differences between categories of the TRG or S system were tested using the Kaplan–Meier (KM) method and log-rank, with the former graded according to College of American Pathologists criteria. The nTRG system was developed by combining the two systems. We compared the discrimination, accuracy, and clinical value of the current American Joint Committee on Cancer (AJCC) post-neoadjuvant staging system (ypTNM) with the nTRG, which integrates TRG and novel metastatic lymph node station number.

**Results:**

In the training cohort, the discrimination, accuracy, and clinical value of nTRG staging groups were better than those of the ypTNM staging groups, which is reflected in the higher C-index and decision curve analysis values. Results were similar in the validation cohort. Multivariate analysis showed that nTRG stage (*P* < 0.001), smoking (*P* = 0.02), and recurrence (*P* < 0.001) were independent prognostic factors.

**Conclusion:**

The nTRG staging system demonstrates superior predictive efficacy to the AJCC ypTNM staging system.

**Supplementary Information:**

The online version contains supplementary material available at 10.1245/s10434-025-18612-y.

Neoadjuvant therapy has become the standard treatment for locally advanced esophageal cancer. However, the primary lesions in patients with esophageal cancer exhibit fibrosis due to inflammation, especially after receiving neoadjuvant chemoradiotherapy (nCRT). The discontinuous and uneven distribution of tumor cells leads to errors in ypT staging, particularly in determining the depth of invasion of the primary lesion after treatment, resulting in an inaccurate prediction of the patient’s prognosis and guidance for sequential treatment.^1^ Therefore, some scholars have used the tumor regression grade (TRG) system to describe the extent of regression of the primary lesion after neoadjuvant therapy.^2–4^ However, the prognostic value of the TRG staging system in neoadjuvant patients remains unclear. In addition, the ypN stage is considered strongly correlated with patients’ prognosis, but lymph node fusion occurs after neoadjuvant therapy, so the number of postoperative lymph node resections cannot be determined in clinical practice. Therefore, some scholars have proposed a staging system based on the number of metastatic lymph node stations (nS).^5–7^ However, the value of the S system in neoadjuvant therapy still needs to be further explored. The aim of this study was to combine the S stage and TRG stage to construct a modified TRG staging system to explore its predictive value following neoadjuvant therapy and to compare the predictive performance of ypTNM staging and modified TRG staging.

## Method

### Study Population

Patients with esophageal squamous cell carcinoma (ESCC) who underwent consecutive esophagectomy after nCRT at West China Hospital of Sichuan University from July 2021 to February 2024 were enrolled in this study. Patients who underwent definitive chemoradiation without subsequent surgery or who were managed with a 'watch-and-wait' approach were excluded from this study cohort. The inclusion criteria were as follows: (1) clinical T1N+M0 or T2-3N0-3M0 locally advanced resectable esophageal cancer; (2) CRT before esophagectomy; and (3) transthoracic esophagectomy and local lymph node dissection. The exclusion criteria were as follows: (1) cancer with invasion of adjacent structures (T4) or distant metastasis (M1); (2) palliative surgery; (3) rescue surgery; (4) coexistence with other malignant tumors; and (5) death within 7 days after the operation. Esophageal and gastroscopy, enhanced computed tomography, endoscopic ultrasonography, bone scanning, magnetic resonance imaging, positron emission tomography, and computed tomography were used for tumor staging during perioperative radical surgery. Neoadjuvant chemotherapy consisted of paclitaxel plus cisplatin or 5-fluorouracil plus cisplatin combined with 45 Gy radiotherapy. Preoperative evaluation determined the feasibility of surgery after 2–4 cycles of the neoadjuvant regimen. Bilateral recurrent laryngeal nerve lymph node metastasis was excluded by color Doppler ultrasound of the cervical lymph nodes in patients who underwent open surgery.

### Surgical Methods

Standard surgical approaches include minimally invasive esophagectomy or thoracotomy. Standard two-field lymphadenectomy was performed; three-field lymphadenectomy was performed only in patients with a high suspicion of cervical nodal disease.

### Pathological Examination

TRG assessment was performed by a single experienced gastrointestinal pathologist specializing in esophageal cancer, Tumor regression was classified strictly according to the detailed histological criteria of the College of American Pathologists^2^: TRG0 (no viable tumor cells), TRG1 (single cell or rare small group of tumor cells), TRG2 (significant residual tumor regression but more than single cell or rare small group of cancer cells), and TRG3 (extensive residual tumor, no significant tumor regression). The pathologist underwent specific training focused on the College of American Pathologists criteria for TRG assessment in esophageal cancer prior to evaluating the study cohort. Pathological staging and lymph node grouping were based on the 8th edition TNM staging of the American Joint Committee on Cancer (AJCC).^3^

### Follow-up

Patients were monitored every 34 months during the first 2 years post-surgery, every 6 months up to 5 years, and annually thereafter. Time to death and recurrence were documented. Overall survival was measured from the date of surgery to death from any cause. Disease-free survival was measured from the date of surgery to the first diagnosis of recurrence.

### Data Analysis

A Cox proportional hazards regression model was applied for both univariate and multivariate analyses. Survival analysis and curve plotting were conducted using the “survival” and “Survminer” packages in R, respectively. Differences between survival curves at 1, 2, and 3 years were assessed using the log-rank test. Patients were randomly assigned to training and validation groups in a 7:3 ratio using R software. The χ^2^ test and Fisher’s exact test compared baseline characteristics between groups. The nomogram was developed based on independent prognostic factors identified through univariate and multivariate logistic regression analyses. Variables with *P* < 0.10 in univariate analysis were included in the multivariate analysis. Model discrimination was evaluated using the Harrell concordance index (C-index), with higher values indicating better differentiation of survival outcomes. Calibration curves were plotted to assess agreement between predicted and actual survival rates. The area under the receiver operating characteristic (ROC) curve (AUC) measures the model’s predictive efficacy. Decision curve analysis (DCA) was performed to determine the clinical utility of the nomogram. All statistical analyses were conducted using SPSS Statistics (version 24, IBM, Armonk, NY, USA) and R (version 3.6.3, Vienna, Austria). Statistical significance was set at a two-sided *P* < 0.05.

## Results

### Patient Characteristics

We screened 514 consecutive patients with ESCC who underwent nCRT followed by esophagectomy at our center between July 2021 and February 2024. The clinicopathological features are summarized in Table 1. After nCRT, 216 patients (42%) achieved TRG0, 77 patients (14.6%) achieved TRG1, 185 patients (36%) achieved TRG2, and 36 patients (7.4%) achieved TRG3.

**Table 1:** Clinicopathological characteristics of patients with esophageal squamous cell carcinoma after neoadjuvant chemoradiotherapy

Characteristics (n=514)	Total
Age	62(56–68)
Sex	
Male	432(84)
Female	82(16)
Location	
Upper	40(7.8)
Middle	324(63)
Lower	150(29.2)
Operation	
Minimal invasive	483(94)
Open	31(6)
Differentiation	
G3	14(3)
G2	188(36.6)
G1	67(13)
Gx	245(47.4)
yT stage	
0	218(42.4)
I	76(14.8)
II	72(14)
III	146(28.4)
IV	2(0.4)
ypN stage	
0	347(68)
I	111(21.6)
II	43(8.4)
III	13(2)
TRG	
TRG0	216(42)
TRG1	77(14.6)
TRG2	185(36)
TRG3	36(7.4)
R0 resection	514(100)
Number of resected LN	23(16-29)
Number of positive LN	0(0-1)

Data are presented as median (interquartile range) or n (%) unless otherwise indicated.

LN, lymph node; TRG, tumor regression grade.

### ypN Stage and TRG Stage Classification Survival Differences

**Fig. 1:**
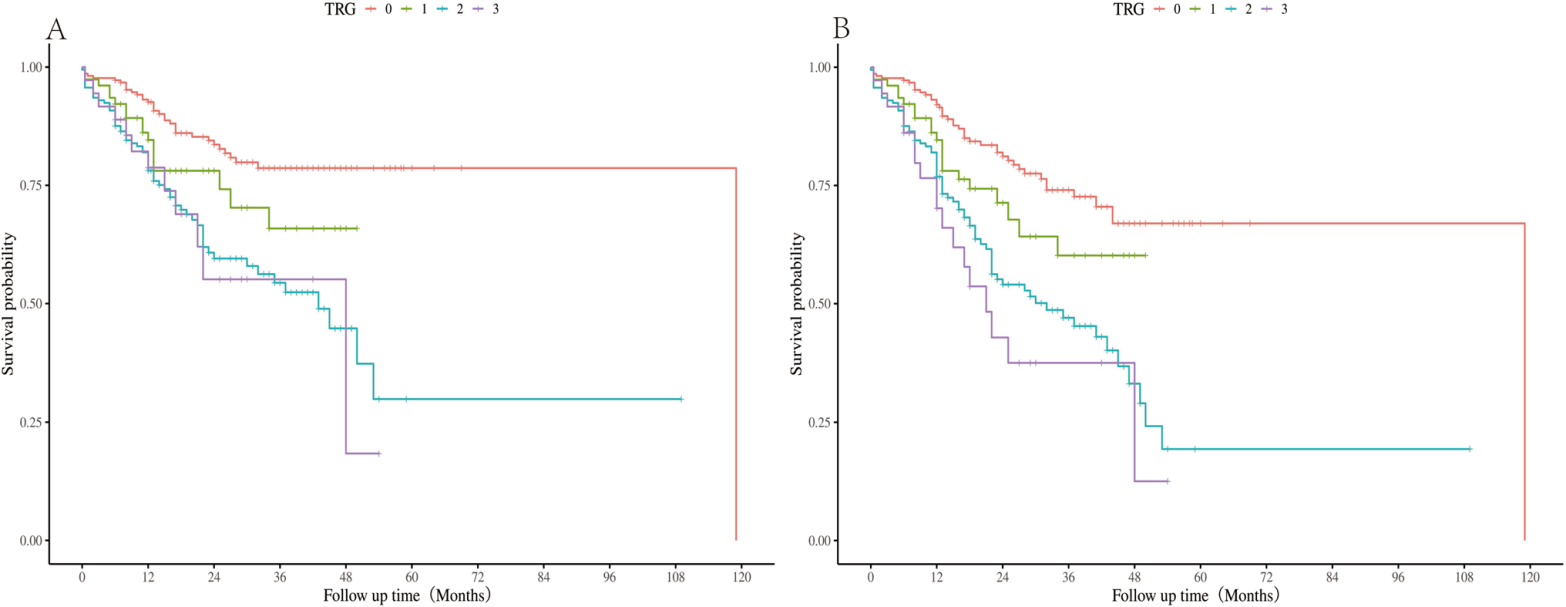
Survival difference in **A** overall survival and **B** disease-free survival according to tumor regression grade (TRG) stage category, *P* < 0.001.

The median follow-up time was 27 months (95% confidence interval [CI] 23.9–6.0). The Kaplan‒Meier survival curves generated from the TRG data are shown in Fig. 1. Kaplan–Meier analysis revealed that the TRG system did not provide optimal stratification of overall survival: (TRG0 vs TRG1: *P*= 0.052; TRG0 vs TRG2: *P* < 0.001; TRG0 vs TRG3: *P* < 0.001; TRG1 vs TRG2: *P*= 0.078; TRG1 vs TRG3: *P*= 0.129; TRG2 vs TRG3: *P*= 0.795) (Fig. 1A). The results were similar when disease-free survival was considered (TRG0 vs TRG1: *P*= 0.065; TRG0 vs TRG2: *P* < 0.001; TRG0 vs TRG3: *P* < 0.001; TRG1 vs TRG2: *P*= 0.04; TRG1 vs TRG3: *P*= 0.011; TRG2 vs TRG3: *P*= 0.252) (Fig. 1B).

**Fig. 2:**
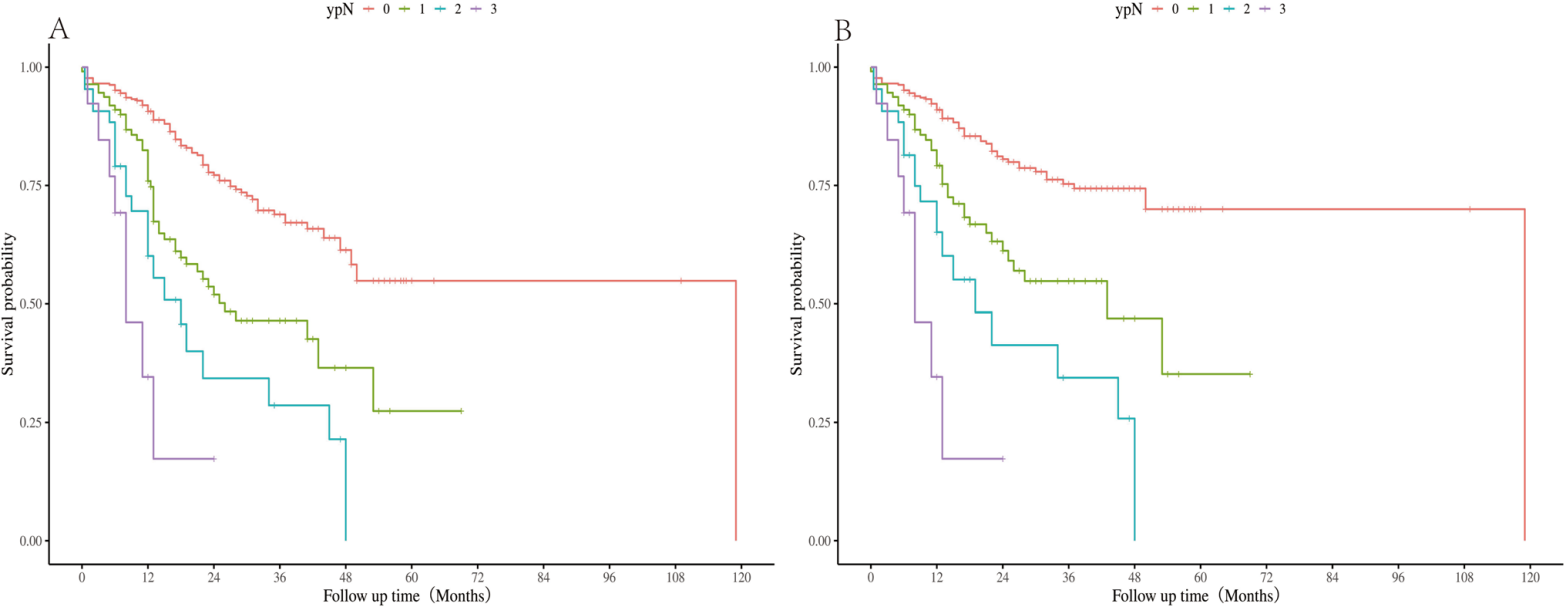
Survival difference of ypN stage categories in **A** overall survival and **B** disease-free survival, *P* < 0.001.

When stratified by lymph node status, there was no significant difference in overall survival (OS) between the ypN2 stage and ypN3 stage in each ypN stage (N0 vs N1: *P* < 0.001; N0 vs N2: *P* < 0.001; N0 vs N3: *P* < 0.001; N1 vs N2: *P*= 0.016; N1 vs N3: *P* < 0.001; N2 vs N3: *P* = 0.128) (Fig. 2A). Results were similar when disease-free survival was considered (N0 vs N1: *P* < 0.001; N0 vs N2: *P* < 0.001; N0 vs N3: *P* < 0.001; N1 vs N2: *P* = 0.020; N1 vs N3: *P* = 0.002; N2 vs N3: *P* = 0.128) (Fig. 2B).

### Value of the S Stage in Neoadjuvant Therapy

**Fig. 3:**
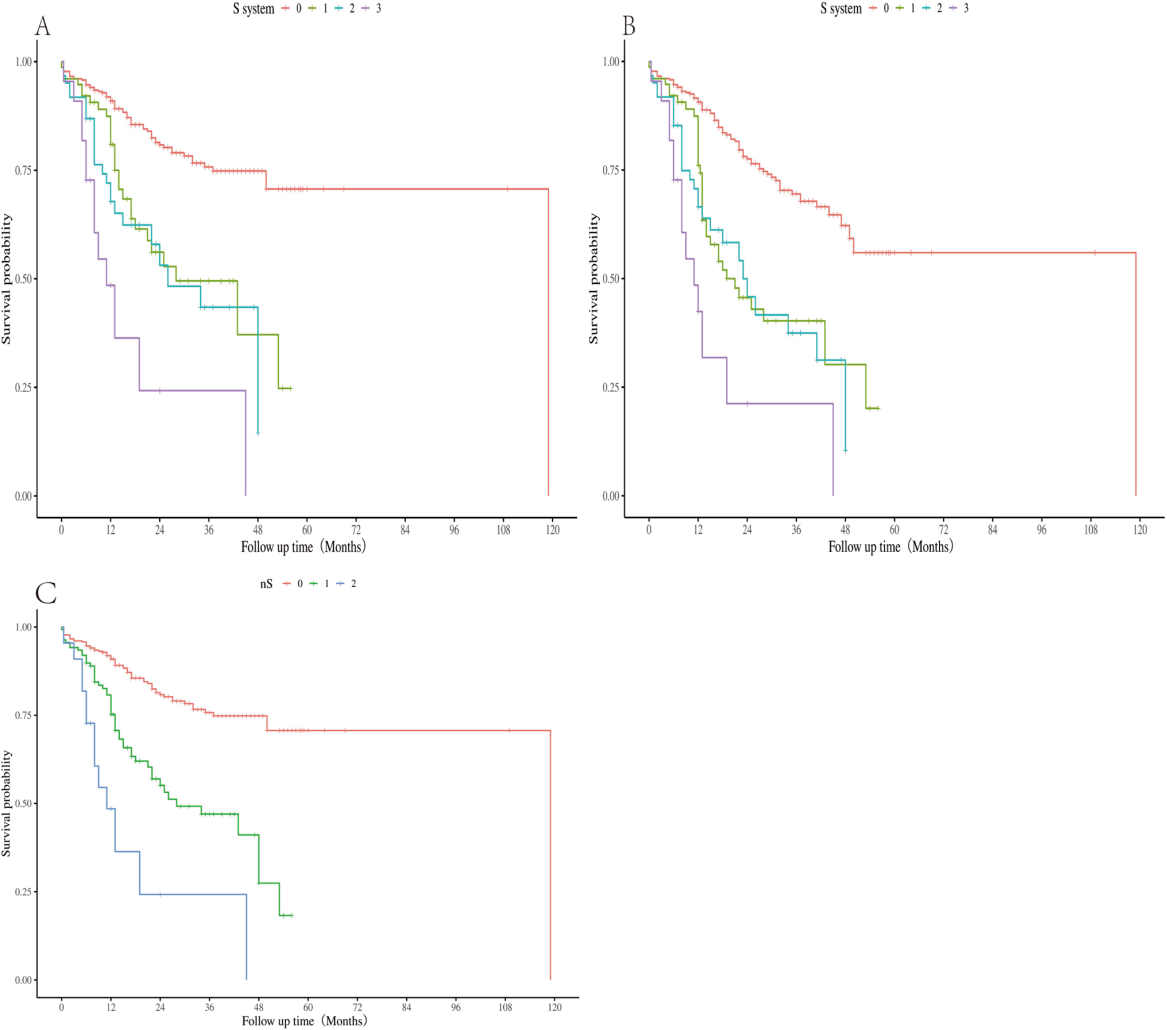
Survival differences in **A** overall survival and **B** disease-free survival among S-stage categories. **C** Survival differences in overall survival by category of nS stage, nS0: 0 metastatic stations; nS1: 1–3 metastatic stations; nS2: >3 metastatic stations.

The Kaplan–Meier curve of OS of the S system showed that there was no significant difference in survival between S1 and S2 stages (hazard ratio [HR] 0.77; 95% confidence interval [CI] 0.45–1.33; *P* = 0.346) (Fig. 3A). The result was also the same in the disease-free survival analysis (HR 0.86; 95% CI 0.52–1.40; *P*= 0.538) (Fig. 3B). Thus, we combined the S1 and S2 staging systems to form a novel S staging system: nS0 (0 station positive lymph nodes); nS1 (positive lymph nodes at stations 1–3); and nS2 (more than three positive lymph nodes). The Kaplan–Meier curve revealed significant differences in survival among the three categories (nS0 vs nS1: *P* < 0.001; nS0 vs nS2: *P* < 0.001; nS1 vs nS2: *P*= 0.003) (Fig. 3C).

### Survival Differences Between ypTNM Stages

**Fig. 4:**
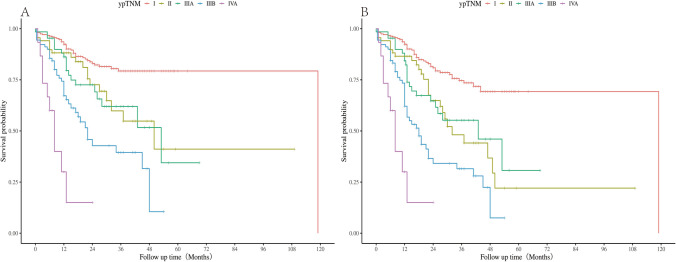
Survival difference of ypTNM classification in **A** overall survival and **B** disease-free survival.

Our results indicated that stages II and IIIA ypTNM in patients after nCRT were not well stratified for OS (II vs IIIA: HR= 0.728; 95% CI 0.47–1.69, *P* = 0.728) (Fig. 4A). Similarly, no difference in disease-free survival was observed between patients with stage II and IIIA disease (II vs IIIA: HR 1.03; 95% CI 0.59–1.80; *P* = 0.918) (Fig. 4B).

### Cox Proportional Hazards Regression Model

ypN stage and nS stage were included in the Cox regression analysis. According to the univariate analysis, TRG was significantly associated with OS (HR 2.47; 95% CI 1.74–3.52; *P* < 0.001). Variables with *P* < 0.10 in the univariate analysis were included in the multivariate analysis. Multivariate analysis revealed that the TRG was not an independent risk factor for OS. ypN stage (HR 1.85; 95% CI 1.13–3.04; *P* = 0.015) and nS stage (HR 2.10; 95% CI 1.38–3.20; *P* < 0.001) were independent prognostic factors for OS (Table 2).

**Table 2:** Cox proportional-hazards model

Variables	Univariate analysis					Multivariate analysis				
	β	SE	Z	*P* value	HR (95% CI)	β	SE	Z	*P* value	HR (95% CI)
T stage										
T0/T1					1.00 (Reference)					1.00 (Reference)
T2/T3/T4	0.91	0.18	5.05	<0.001	2.49 (1.75–3.55)	0.53	0.30	1.75	0.080	1.69 (0.94–3.06)
N stage										
N0/N1					1.00 (Reference)					1.00 (Reference)
N2/N3	1.40	0.21	6.52	<0.001	4.05 (2.66–6.16)	0.62	0.25	2.42	0.015	1.85 (1.13–3.04)
Location										
Lower					1.00 (Reference)					
Upper/middle	0.09	0.20	0.45	0.655	1.09 (0.74–1.60)					
Sex										
Female					1.00 (Reference)					1.00 (Reference)
Male	0.73	0.29	2.50	0.012	2.08 (1.17–3.69)	0.44	0.30	1.47	0.141	1.55 (0.86–2.79)
Differentiation										
G1/G2					1.00 (Reference)					1.00 (Reference)
G3/Gx	-0.40	0.18	-2.26	0.024	0.67 (0.47–0.95)	0.28	0.22	1.28	0.200	1.32 (0.86–2.01)
nS stage										
nS0					1.00 (Reference)					1.00 (Reference)
nS1/2	1.20	0.18	6.73	<0.001	3.30 (2.33–4.68)	0.74	0.21	3.46	<0.001	2.10 (1.38–3.20)
TRG										
TRG0/1					1.00 (Reference)					1.00 (Reference)
TRG2/3	0.91	0.18	5.01	<0.001	2.47 (1.74–3.52)	0.29	0.31	0.95	0.345	1.34 (0.73–2.47)

CI, confidence interval; HR, hazard ratio; SE, standard error; TRG, tumor regression grade.

### Establishment of a Novel Tumor Regression Grading System

We developed a novel TRG system by combining the TRG with the novel number of metastatic nS. The stages include the nI stage (TRG0-1nS0), nII stage (TRG2-3nS0), nIII stage (TRG0-1nS1 or TRG2-3nS1), and nIV stage (TRG0-3nS2). The novel TRG system stratified the survival curves well (nI vs nII: *P*= 0.03; nI vs nII: *P* < 0.001; nI vs nIV: *P* < 0.001; nII vs nIII: *P*= 0.04; nII vs nIV: *P* < 0.001; nIII vs nIV: *P*= 0.03) (Supplement Fig. 1A). Results were similar for disease-free survival (nI vs nII: *P* < 0.001; nI vs nII: *P* < 0.001; nI vs nIV: *P* < 0.001; nII vs nIII: *P*= 0.03; nII vs nIV: *P* < 0.001; nIII vs nIV: *P*= 0.04) (Supplement Fig. 1B).

### Evaluation of the Predictive Performance of the Model

The enrolled patients were randomly assigned to a training set (70%) and a validation set (30%). No difference in balance was observed between the training and validation sets (Table 3). We included eight covariates, including nTRG stage, sex, postoperative adjuvant therapy, recurrence, pathological complete response (pCR), location, smoking status, and tumor differentiation, into the regression analysis. Covariates with *P* < 0.1 were included in the multivariate analysis. Univariate Cox regression analysis revealed that nTRG, sex, postoperative adjuvant therapy, recurrence, pCR, smoking status, and tumor differentiation were associated with OS (*P* < 0.1), and multivariate Cox regression analysis was subsequently performed. Three variables were identified as independent prognostic factors: recurrence, smoking status, and nTRG (Supplement Table 1). Based on the screened independent prognostic factors, a nomogram was established to predict the 12-, 24-, and 36-month OS of patients with ESCC after nCRT (Supplement Fig. 2). The C-index of the nomogram model was 0.73 (95% CI 0.67–0.78) in the training set and 0.72 (95% CI 0.616–0.805) in the validation set, indicating that the prediction model had sufficient discrimination power. The ROC curve revealed that the AUCs of the model at 12, 24, and 36 months were 0.76, 0.81, and 0.82, respectively, in the training set and 0.72, 0.72, and 0.69, in the validation set, indicating that the model had good accuracy (Supplement Fig. 3). Additionally, the calibration curves of the training set and the validation set at 12, 24, and 36 months demonstrated high consistency between the actual observations and the nomogram prediction results (Supplement Fig. 4).

**Table 3:** Balance test between training set and validation set

Variables	Total (n = 514)	test (n = 155)	train (n = 359)	Statistic	*P* value
T stage				χ^2^=1.39	0.846
0	218 (42.41)	62 (40.00)	156 (43.45)		
I	76 (14.79)	23 (14.84)	53 (14.76)		
II	72 (14.01)	25 (16.13)	47 (13.09)		
III	146 (28.40)	44 (28.39)	102 (28.41)		
IV	2 (0.39)	1 (0.65)	1 (0.28)		
TRG				χ^2^=0.72	0.398
TRG0/1	293 (57.00)	84 (54.19)	209 (58.22)		
TRG2/3	221 (43.00)	71 (45.81)	150 (41.78)		
N stage				χ^2^=1.63	0.653
0	347 (67.51)	103 (66.45)	244 (67.97)		
I	111 (21.60)	33 (21.29)	78 (21.73)		
II	43 (8.37)	13 (8.39)	30 (8.36)		
III	13 (2.53)	6 (3.87)	7 (1.95)		
ypTNM				χ^2^=4.66	0.324
I	276 (53.70)	79 (50.97)	197 (54.87)		
II	68 (13.23)	23 (14.84)	45 (12.53)		
IIIA	65 (12.65)	19 (12.26)	46 (12.81)		
IIIB	90 (17.51)	26 (16.77)	64 (17.83)		
IVA	15 (2.92)	8 (5.16)	7 (1.95)		
Location				χ^2^=2.34	0.126
Lower	150 (29.18)	38 (24.52)	112 (31.20)		
Upper/middle	364 (70.82)	117 (75.48)	247 (68.80)		
Differentiation				χ^2^=1.86	0.602
G1	14 (2.72)	6 (3.87)	8 (2.23)		
G2	188 (36.58)	55 (35.48)	133 (37.05)		
G3	67 (13.04)	23 (14.84)	44 (12.26)		
Gx	245 (47.67)	71 (45.81)	174 (48.47)		
Sex				χ^2^=0.36	0.551
Female	82 (15.95)	27 (17.42)	55 (15.32)		
Male	432 (84.05)	128 (82.58)	304 (84.68)		
Recurrence				χ^2^=0.26	0.607
No	431 (83.85)	128 (82.58)	303 (84.40)		
Yes	83 (16.15)	27 (17.42)	56 (15.60)		
pCR				χ^2^=1.08	0.298
No	331 (64.40)	105 (67.74)	226 (62.95)		
Yes	183 (35.60)	50 (32.26)	133 (37.05)		
POAT				χ^2^=1.78	0.182
No	275 (53.50)	76 (49.03)	199 (55.43)		
Yes	239 (46.50)	79 (50.97)	160 (44.57)		
Smoking				χ^2^=0.00	0.950
No	241 (46.89)	73 (47.10)	168 (46.80)		
Yes	273 (53.11)	82 (52.90)	191 (53.20)		
nS stage				χ^2^=1.28	0.527
0	355 (69.07)	106 (68.39)	249 (69.36)		
1	137 (26.65)	40 (25.81)	97 (27.02)		
2	22 (4.28)	9 (5.81)	13 (3.62)		

Data are presented as median (interquartile range) or n (%) unless otherwise indicated.

pCR, pathological complete response; POAT, postoperative adjuvant therapy; TRG, tumor regression grade; χ^2^, chi-squared test

### Comparison Between the nTRG System and the ypTNM System

The time-dependent ROC curve revealed that the discriminative power of the nTRG staging system was better than that of the AJCC TNM staging system in patients with locally advanced esophageal cancer in both the training set (AUC 71.88 vs 68.97) and the validation set (AUC 71.88 vs 68.97) (Supplement Figure 5). DCA demonstrated an improved net benefit of the nTRG model compared with the conventional ypTNM staging system, with a wide range of threshold probabilities (Supplement Fig. 6). In the accuracy analysis of the Net Reclassification Index （NRI）, the nTRG still outperformed the ypTNM staging system (Supplement Table 2). In the training set, the 12-, 24-, and 36-month NRIs of nTRG were 0.111, 0111, and 0.006, respectively. In the validation set, the 12-, 24-, and 36-month NRIs of the nomogram were 0.257, 0.128, and 0.058, respectively. There was a significant difference at 12 months.

## Discussion

This study demonstrates the independent prognostic value of nTRG, as confirmed by both univariate and multivariate analyses. A model incorporating nTRG was constructed to predict patient survival at 1, 2, and 3 years, confirming the model's discrimination and accuracy. More importantly, the nTRG system is superior to the ypTNM classification in reflecting treatment response and predicting patient survival. TRG or ypn classification alone did not adequately predict patient survival. However, TRG in this study was statistically significant in the univariate survival analysis but not a predictive factor in the multivariate analysis, which was consistent with some of the results of previous studies.^8,9^ This may be related to the fact that all the enrolled patients underwent R0 resection, thus reducing the impact of the primary lesion on survival. Furthermore, the role of TRG within the nTRG system is not that of an isolated predictor but rather a modifier that refines risk stratification based on lymph node status. TRG quantifies the primary tumor's response to neoadjuvant therapy, whereas the nS stage reflects the burden of nodal disease. These two components represent distinct biological dimensions of treatment efficacy. Although TRG lost its independent significance in the multivariate analysis, likely due to collinearity with nodal status, its strong correlation with survival in the univariate analysis (HR 2.47; *P* < 0.001) confirms its intrinsic prognostic value. The integration of TRG with nS in the nTRG system creates synergistic interaction, identifying key risk subgroups that neither system alone could capture effectively: TRG0-1 + nS1 (nIII): good initial response but residual nodal involvement → intermediate risk. TRG2-3 + nS0 (nII): worse primary response, no lymph node metastasis → worse than nI (*P* = 0.03). This explains the superiority of nTRG over two independent TRGs (*P* < 0.001 vs. *P* = 0.795 for TRG2/3) and ypN stage (C-index 0.72 vs. 0.68). In terms of biological plausibility, primary TRG and lymph node response are mechanistically linked, but not the same process, which are associated with poorer survival. TRG-driven fibrosis may obscure residual micro-metastases, making nodal status alone insufficient for risk assessment. To improve the prediction accuracy of the TRG in neoadjuvant therapy, Yun et al.^11^ combined the Schneider TRG system with ypN staging to construct a novel staging system to predict patient survival prognosis.^10^ In contrast, Wong et al.^13^ combined the Becker TRG system with lymph node status to evaluate patient prognosis.^12^ Although both studies effectively demonstrated that the combined staging system not only has significant survival differences but also has greater predictive performance than the traditional ypTNM staging system, 8th edition. However, the TRG staging system used in this study is based on the quantification of the fraction of residual tumor cells after treatment. Spontaneous necrosis and fibrosis following tumor necrosis can lead to errors in assessment, which can vary significantly between evaluators after treatment. Both the Yun and the Wong models depend on the number of lymph nodes (ypN), but lymph node fusion can lead to counting bias in clinical practice. nTRG uses the number of metastatic nS, which avoids the interference of fusion and is more consistent with the actual postoperative pathological evaluation. The model by Yun et al.^11^ combined TRG0-1 and ypN0 into the low-risk group. However, nTRG did not distinguish between patients with TRG2-3 and nS0 by independently classifying TRG2-3 as nII stage, which significantly improved the risk identification ability for this population. Among them, nS staging is based on the AJCC definition of lymph node stations, without relying on microscopic count, and the consistency of pathological reports is higher.

Lymph node status has been proven to be an independent prognostic factor for patients with esophageal cancer treated with nCRT combined with surgery.^14^ The MAGIC trial by Smyth et al.^15^ revealed that lymph node stage status is an independent predictor of OS in patients with esophageal cancer after neoadjuvant chemotherapy. In addition, Miyata et al.^8^ noted that the number of positive lymph nodes, but not the TRG, is a reliable predictor of ESCC. However, when stratified by lymph node status in this study, there was no significant difference in OS between the ypN2 stage and ypN3 stage in each ypN stage (HR 0.46; 95% CI 0.1–1.07; *P*= 0.128). This finding is similar to the results of Peng et al.^16^ and Gu et al.^17^ This may be because the lymph node response of patients with ESCC after nCRT and the response of the primary lesion may not be parallel. In addition, in clinical practice, lymph nodes are sometimes fragmented into multiple pieces, or several lymph nodes are fused into a large lymph node during the operation; therefore, it is impossible to count the number of metastatic lymph nodes accurately. Besides, skipping lymph node metastasis has also been shown to be negatively correlated with prognosis,^18^ but TNM staging cannot effectively distinguish it. Although the primary tumor may be highly reactive, the TRG system for evaluating the response of the primary tumor may no longer have a high prognostic value for patients with advanced disease who still have lymph node metastasis after neoadjuvant therapy.

A study by Yuan et al.^19^ on the prognostic value of the number of metastatic lymph node stations revealed that, compared with N staging, which is based on the number of metastatic lymph nodes, S staging, which is based on the number of metastatic lymph node stations, also has a comparable prognostic value in patients with esophageal cancer (C-index: 0.659 vs 0.658). However, this study did not confirm the application value of S staging in neoadjuvant patients.^19^ Therefore, we constructed a novel S staging system: nS0, nS1, nS2, and combined it with the TRG system to explore a novel TRG system. The novel TRG staging system has good survival stratification among different subtypes, and its prognostic value and efficacy are comparable to those of the AJCC ypTNM staging system. Therefore, nTRG is helpful for postoperative treatment decisions. Patients with nI stage have a better prognosis. De-escalation of adjuvant therapy may be considered to minimize treatment-related toxicity, especially in older patients or those with coexisting conditions, and less frequent surveillance may suffice given the lower recurrence risk. Patients at high risk for recurrence in the nIII/nIV stage may require intensified adjuvant therapy, and intensified surveillance is justified to detect early recurrence, with salvage therapy still feasible. Pathological reports should routinely include ypTNM and nTRG stages in the clinical workflow of esophageal cancer treatment. When ypTNM indicates intermediate risk and nTRG indicates high risk, the multidisciplinary oncology committee can take advantage of the characteristics of nTRG to resolve the differences in treatment recommendations. However, when patients with nIV disease need to discuss prognosis and palliative care options clearly, nTRG can be used to guide patient consultation.

Consistent with previous studies, patients who experienced pCR in this study had a relatively superior survival prognosis (Supplementary Fig. 7).^20,21^ In this study, pCR was not an independent prognostic factor for patients; this may be because of the inclusion of TRG0 and nS0 in phase I of nTRG. It may also be because CRT plus cisplatin yields a higher pCR rate than the CROSS regimen.^22^

Our cohort represents a surgically resectable population, which introduces potential selection bias. Patients achieving clinical complete response after nCRT may avoid surgery, whereas non-responders with progressive disease may become inoperable. Our study excluded both extremes, potentially narrowing the prognostic spectrum. However, using pathological parameters to improve postoperative risk stratification aligns with our expectations. The exclusion of non-surgical patients likely skewed our cohort toward intermediate responders; this may attenuate survival differences between staging groups but would not invalidate the comparative superiority of nTRG over ypTNM within the operable population. Notably, our cohort included patients with poor regression (TRG3: 7.4%) and advanced nodal disease (nS2: 4.3%), preserving critical prognostic heterogeneity. In the validation study by Wong et al.,^13^ a similar surgical cohort design was also adopted, as pathological assessment requires the removal of samples. Our research results are only applicable to the specific population of surgical patients, which is precisely the target population of this staging system.^13^

However, our study has several limitations. First, this study is retrospective and from a single center, so the sample size needs to be further expanded to ensure its validity. Another limitation of our study is that since TRG only evaluated tumor invasion into the esophageal submucosa but not primary tumor size reduction before and after neoadjuvant therapy, a retrospective study found that the rate of tumor volume reduction before and after nCRT was an independent prognostic factor for PFS and therefore affected the optimal timing of surgery after neoadjuvant therapy.^23,24^ From the perspective of selecting variables based on methodology, we also have some limitations. While our model prioritized statistically significant variables, future studies may consider including key clinical covariates by force to validate their adjusted effects. Our study spanned from July 2021 to February 2024. The relatively short time interval is not sufficient to prove the value of nTRG on long-term survival, and further follow-up is needed to verify the predictive value of nTRG. In addition, despite our internal validation, there is still a lack of external cohort validation to demonstrate the reproducibility of our experiments. Third, our cohort does not represent the full spectrum of nCRT recipients, as it excludes patients who are not undergoing non-surgical treatment. This selection bias may limit the generalizability of the findings to all patients with locally advanced ESCC. Future multicenter studies incorporating clinical staging data from non-surgical cohorts are warranted.

In addition, this study did not compare lymph node downstaging before and after neoadjuvant therapy, and a meta-analysis of 14 studies suggested that lymph node downstaging predicts better survival.^25^

## Conclusion

Our results revealed that the novel TRG system had significant survival differences compared with the ypTNM classification, and the clinical accuracy, discrimination, and prediction efficiency of the two models were comparable. The novel TRG system is helpful for postoperative treatment decision-making and survival monitoring.

## Supplementary Information

Below is the link to the electronic supplementary material.

Supplementary file1 (Fig. 1 Survival differences in overall survival according to nTRG stage category (A). Survival differences in disease-free survival according to nTRG stage category (B).nI: TRG0-1 + nS0nII; TRG2;3 + nS0;nIII: TRG0-1 + nS1 or TRG2-3 + nS1;nIV: TRG0-3 + nS2)

Supplementary file2 (Fig. 2 The nomogram including nTRG staging to predict the OS of patients after nCRT at 12, 24, and 36 months)

Supplementary file3 (Fig. 3 AUC values of the model at 12, 24, and 36 months in the training and validation sets)

Supplementary file4 (Fig. 4 Calibration curves for 12 ~ (A), 24 ~ (B), 36 months (C), OS for the training set and 12 ~ (D), 24 ~ (E), 36 months (F) OS for the validation set. The dashed line indicates a good match between the nomogram prediction (X-axis) and the actual survival outcome (Y-axis). The closer the dot is to the dashed line, the higher the prediction accuray)

Supplementary file5 (Fig. 5 Time-dependent ROC curve comparison of OS in the training set (A) and validation set (B) for nTRG stage and ypTNM stage)

Supplementary file6 (Fig. 6 DCA was used to predict 12-month (A), 24-month (B), 36-month (C), and OS in the training set, and DCA was used to predict 12-month (D), 24-month (E), and 36-month (F) OS in the validation set)

Supplementary file7 (Fig. 7 Survival difference between patients with and without PCR)

Supplementary file8 (DOCX 25 kb)

Supplementary file9 (DOCX 12 kb)

## Data Availability

The datasets generated and/or analyzed during the current study are available from the corresponding author on reasonable request.
