# Supplementary figures and images for: Prognostic Value of a Novel Staging System Integrating Lymph Node Station Number and Tumor Regression Grade for Esophageal Cancer Following Neoadjuvant Chemoradiotherapy
Source: Ann Surg Oncol. 2025 Nov 9;33(2):996–1006. doi: 10.1245/s10434-025-18612-y (PMC12765742; doi:10.1245/s10434-025-18612-y)

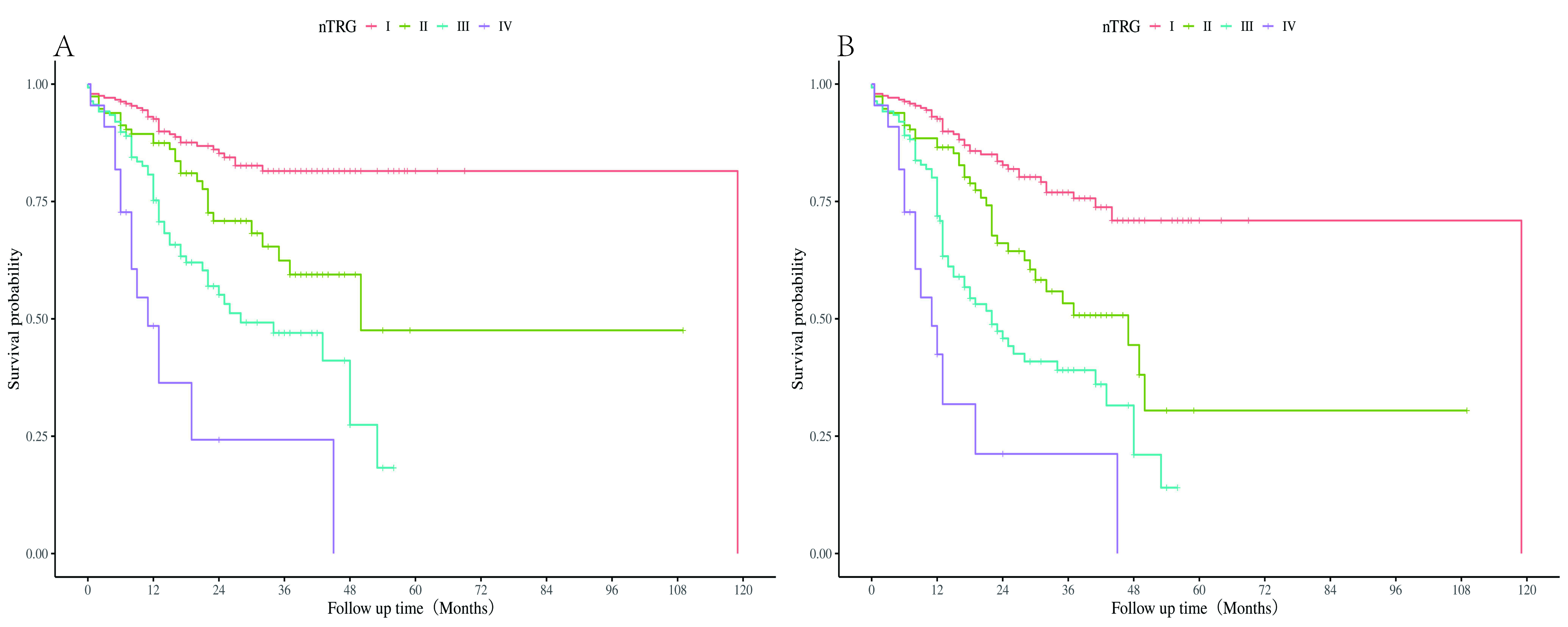

Supplement: Supplementary file 1 — Supplementary file1 (Fig. 1 Survival differences in overall survival according to nTRG stage category (A). Survival differences in disease-free survival according to nTRG stage category (B).nI: TRG0-1 + nS0nII; TRG2;3 + nS0;nIII: TRG0-1 + nS1 or TRG2-3 + nS1;nIV: TRG0-3 + nS2) [file 10434_2025_18612_MOESM1_ESM.tif]

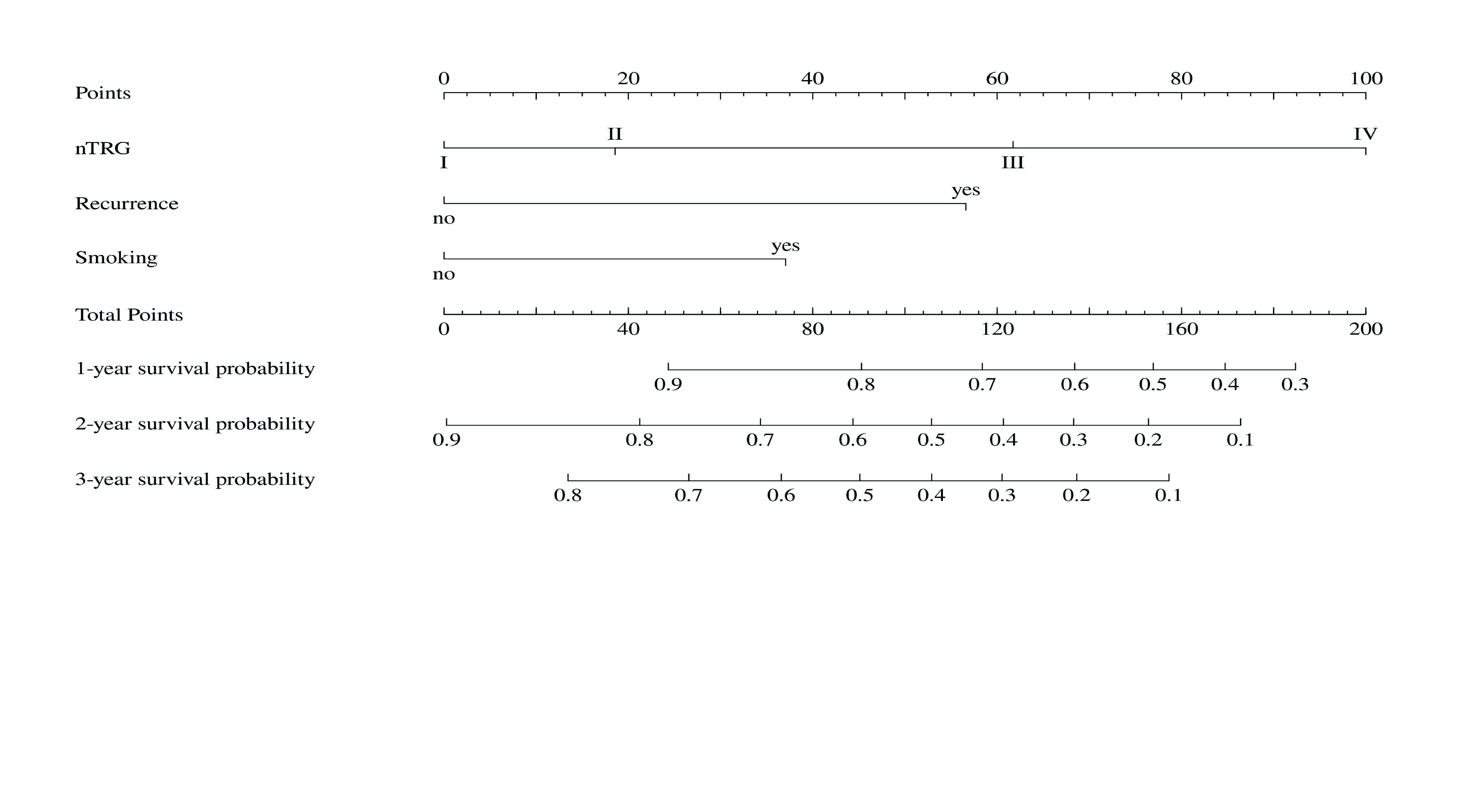

Supplement: Supplementary file 2 — Supplementary file2 (Fig. 2 The nomogram including nTRG staging to predict the OS of patients after nCRT at 12, 24, and 36 months) [file 10434_2025_18612_MOESM2_ESM.tif]

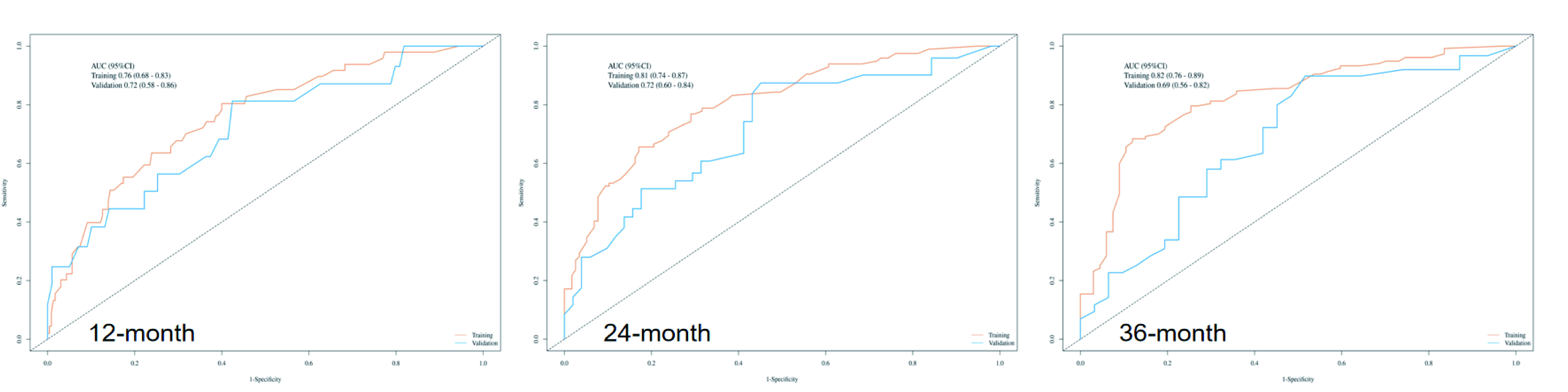

Supplement: Supplementary file 3 — Supplementary file3 (Fig. 3 AUC values of the model at 12, 24, and 36 months in the training and validation sets) [file 10434_2025_18612_MOESM3_ESM.tif]

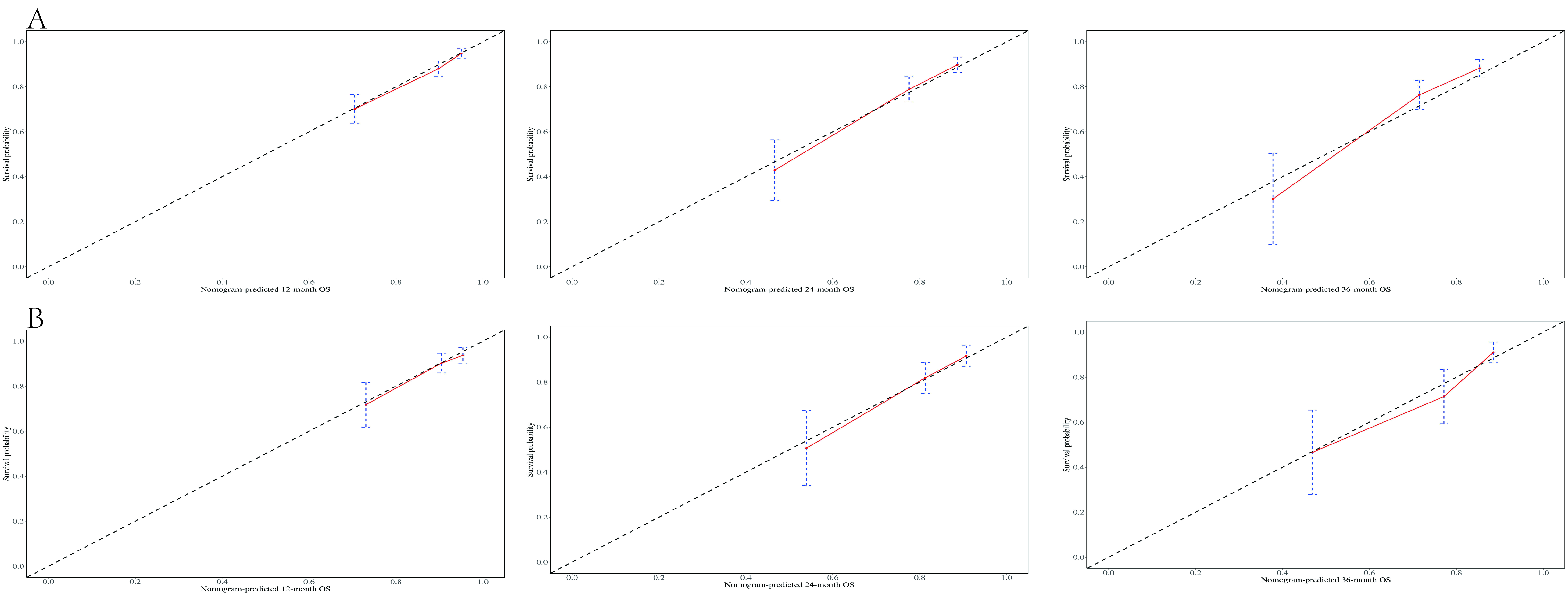

Supplement: Supplementary file 4 — Supplementary file4 (Fig. 4 Calibration curves for 12 ~ (A), 24 ~ (B), 36 months (C), OS for the training set and 12 ~ (D), 24 ~ (E), 36 months (F) OS for the validation set. The dashed line indicates a good match between the nomogram prediction (X-axis) and the actual survival outcome (Y-axis). The closer the dot is to the dashed line, the higher the prediction accuray) [file 10434_2025_18612_MOESM4_ESM.tif]

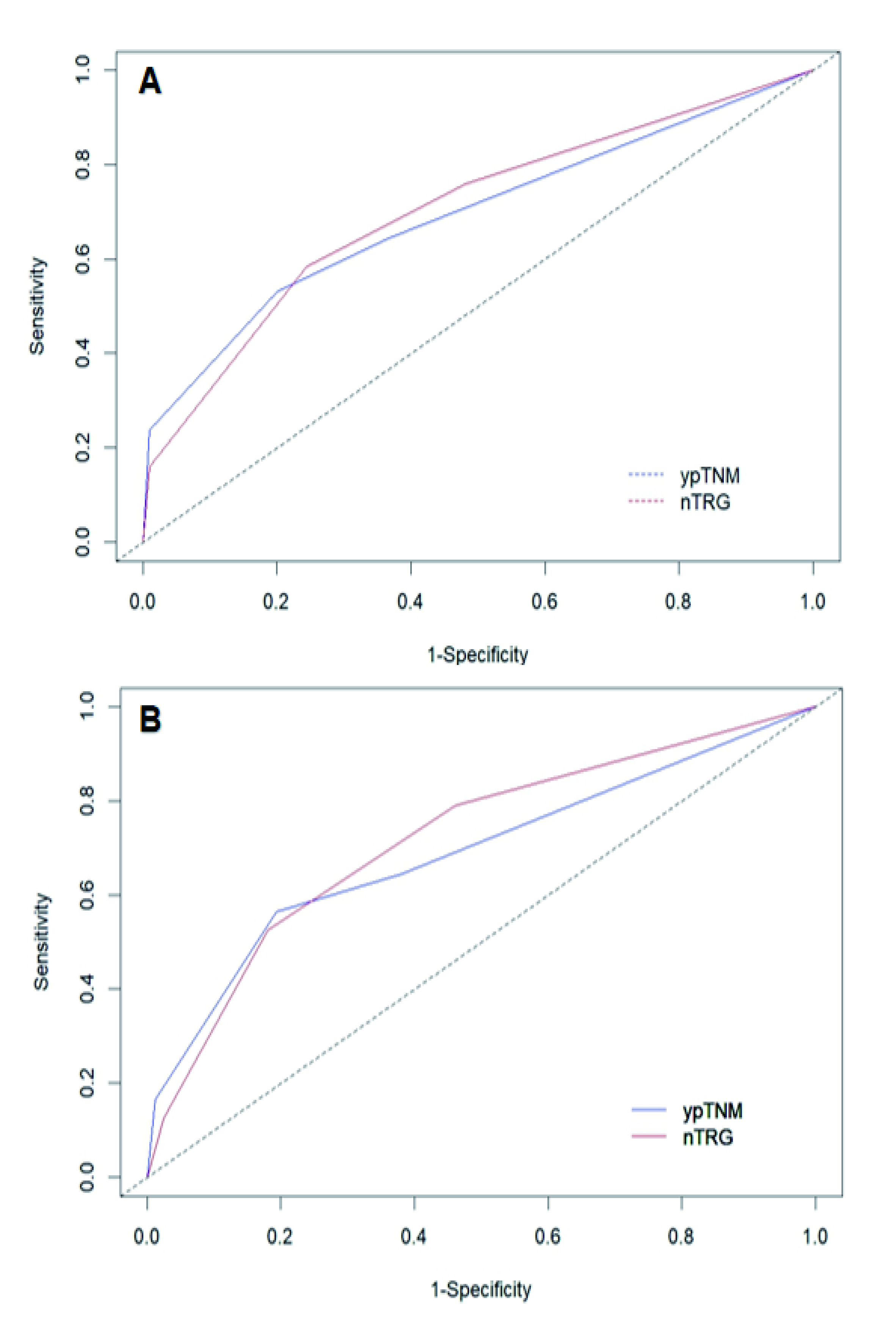

Supplement: Supplementary file 5 — Supplementary file5 (Fig. 5 Time-dependent ROC curve comparison of OS in the training set (A) and validation set (B) for nTRG stage and ypTNM stage) [file 10434_2025_18612_MOESM5_ESM.tif]

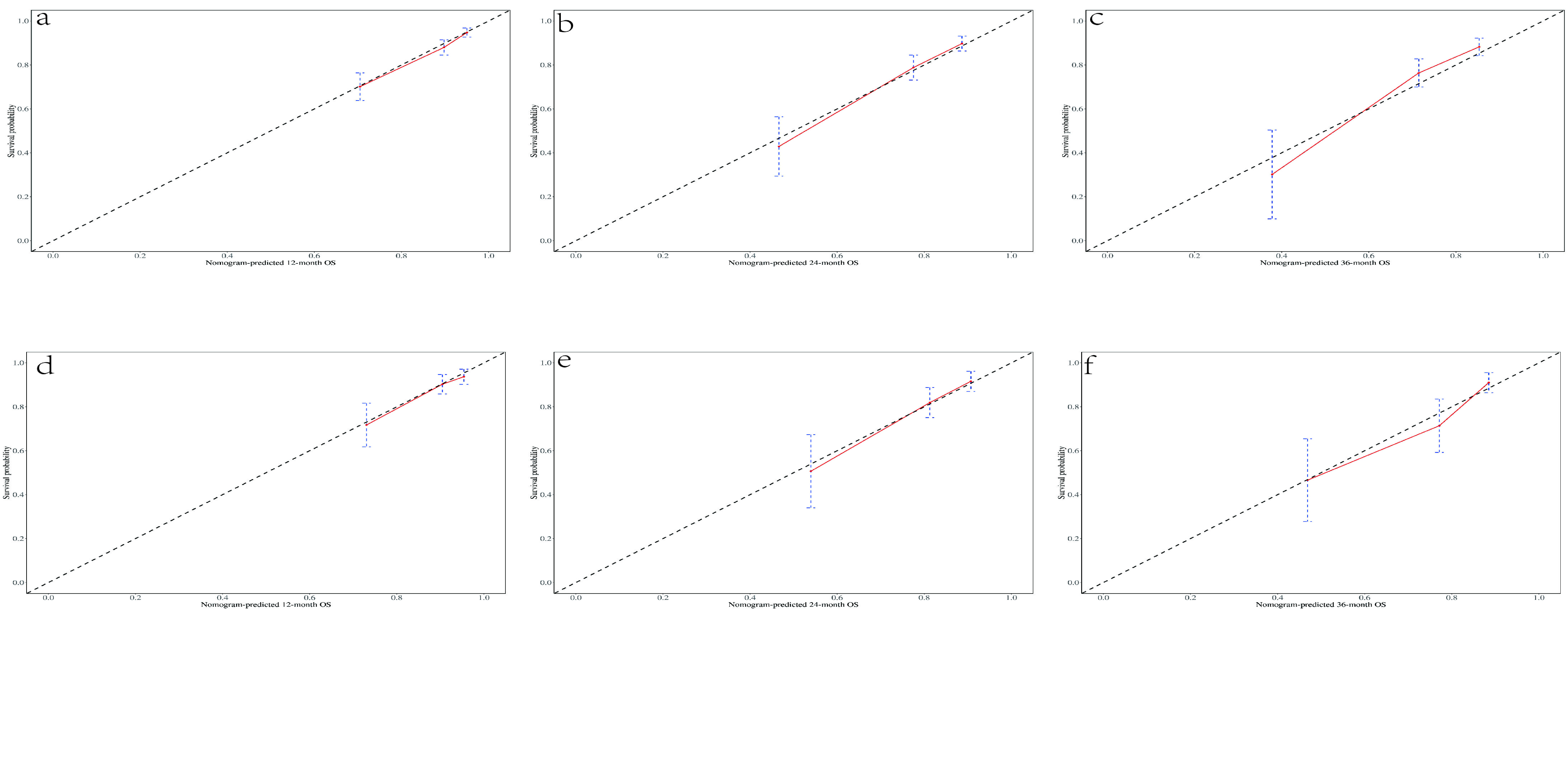

Supplement: Supplementary file 6 — Supplementary file6 (Fig. 6 DCA was used to predict 12-month (A), 24-month (B), 36-month (C), and OS in the training set, and DCA was used to predict 12-month (D), 24-month (E), and 36-month (F) OS in the validation set) [file 10434_2025_18612_MOESM6_ESM.tif]

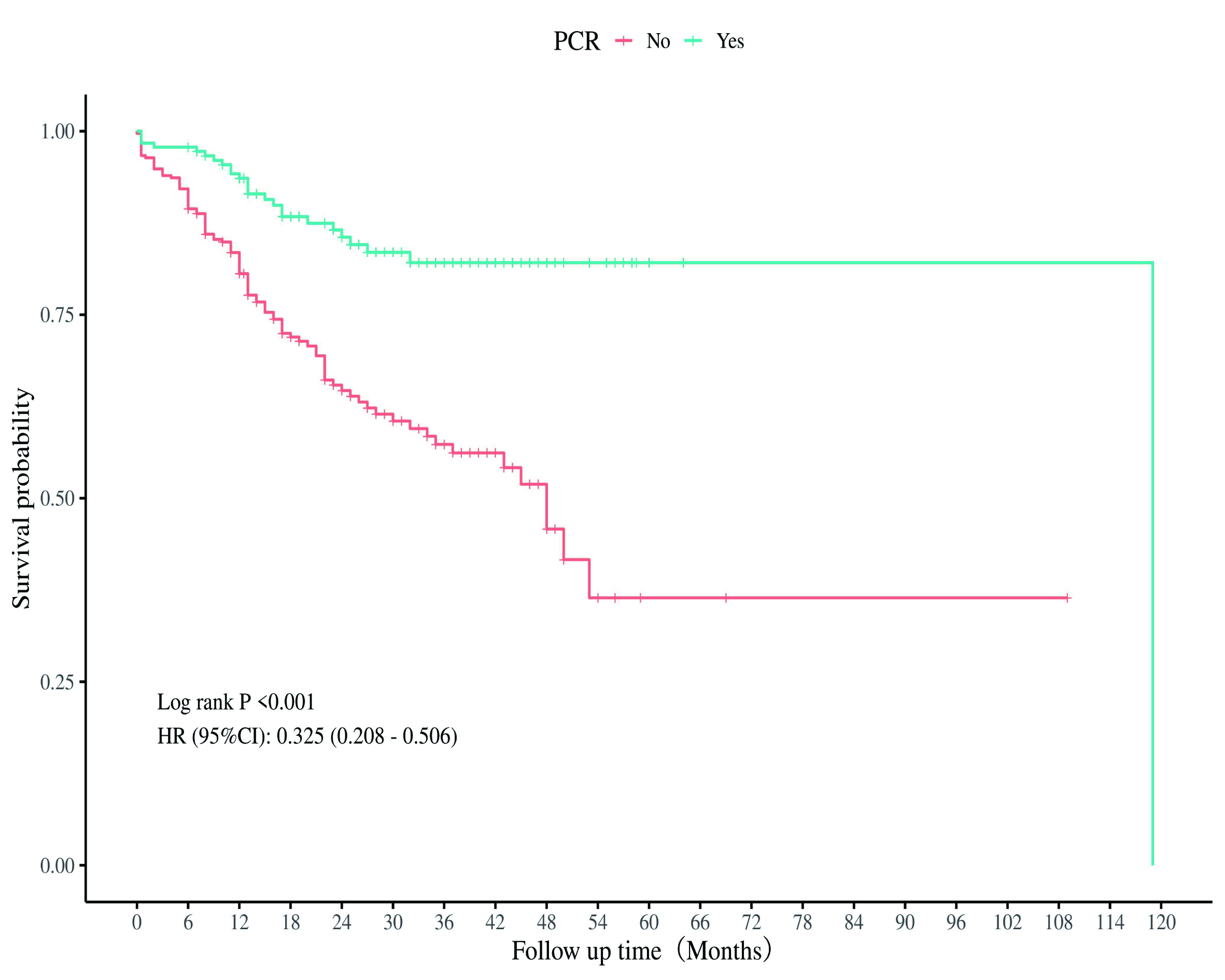

Supplement: Supplementary file 7 — Supplementary file7 (Fig. 7 Survival difference between patients with and without PCR) [file 10434_2025_18612_MOESM7_ESM.tif]
