# Supplementary material for: Prognostic Value of a Novel Staging System Integrating Lymph Node Station Number and Tumor Regression Grade for Esophageal Cancer Following Neoadjuvant Chemoradiotherapy
Source: Ann Surg Oncol. 2025 Nov 9;33(2):996–1006. doi: 10.1245/s10434-025-18612-y (PMC12765742; doi:10.1245/s10434-025-18612-y)
Supplement: Supplementary file 8 — Supplementary file8 (DOCX 25 kb) [file 10434_2025_18612_MOESM8_ESM.docx]

### Supplementary Table1: Cox proportional hazards model including nTRG stage

| Variables | Univariate analysis | | | | |  | Multivariate analysis | | | | |
| --- | --- | --- | --- | --- | --- | --- | --- | --- | --- | --- | --- |
|  | β | S.E | Z | *P* | hR (95%CI) |  | β | S.E | Z | *P* | hR (95%CI) |
| nTRG |  |  |  |  |  |  |  |  |  |  |  |
| I |  |  |  |  | 1.00 (Reference) |  |  |  |  |  | 1.00 (Reference) |
| II | 0.74 | 0.25 | 2.93 | **0.003** | 2.10 (1.28 ~ 3.44) |  | 0.54 | 0.25 | 2.11 | **0.034** | 1.71 (1.04 ~ 2.82) |
| III | 1.37 | 0.22 | 6.13 | **<.001** | 3.92 (2.53 ~ 6.06) |  | 1.13 | 0.23 | 4.97 | **<.001** | 3.10 (1.99 ~ 4.85) |
| IV | 2.26 | 0.33 | 6.79 | **<.001** | 9.61 (5.00 ~ 18.48) |  | 1.87 | 0.34 | 5.51 | **<.001** | 6.51 (3.34 ~ 12.67) |
| Location |  |  |  |  |  |  |  |  |  |  |  |
| Lower |  |  |  |  | 1.00 (Reference) |  |  |  |  |  |  |
| Upper/Middle | 0.09 | 0.20 | 0.45 | 0.655 | 1.09 (0.74 ~ 1.60) |  |  |  |  |  |  |
| Recurrence |  |  |  |  |  |  |  |  |  |  |  |
| no |  |  |  |  | 1.00 (Reference) |  |  |  |  |  | 1.00 (Reference) |
| yes | 1.34 | 0.18 | 7.42 | **<.001** | 3.82 (2.68 ~ 5.44) |  | 1.05 | 0.19 | 5.66 | **<.001** | 2.85 (1.98 ~ 4.11) |
| Gender |  |  |  |  |  |  |  |  |  |  |  |
| Female |  |  |  |  | 1.00 (Reference) |  |  |  |  |  |  |
| Male | 0.73 | 0.29 | 2.50 | **0.012** | 2.08 (1.17 ~ 3.69) |  |  |  |  |  |  |
| PCR |  |  |  |  |  |  |  |  |  |  |  |
| no |  |  |  |  | 1.00 (Reference) |  |  |  |  |  |  |
| yes | -1.13 | 0.23 | -4.96 | **<.001** | 0.32 (0.21 ~ 0.51) |  |  |  |  |  |  |
| POAT |  |  |  |  |  |  |  |  |  |  |  |
| no |  |  |  |  | 1.00 (Reference) |  |  |  |  |  |  |
| yes | 0.40 | 0.18 | 2.24 | **0.025** | 1.49 (1.05 ~ 2.12) |  |  |  |  |  |  |
| Smoking |  |  |  |  |  |  |  |  |  |  |  |
| no |  |  |  |  | 1.00 (Reference) |  |  |  |  |  | 1.00 (Reference) |
| yes | 0.67 | 0.19 | 3.54 | **<.001** | 1.94 (1.35 ~ 2.81) |  | 0.58 | 0.19 | 3.06 | **0.002** | 1.78 (1.23 ~ 2.59) |
| Differentiation |  |  |  |  |  |  |  |  |  |  |  |
| G1/G2 |  |  |  |  | 1.00 (Reference) |  |  |  |  |  |  |
| G3/Gx | -0.40 | 0.18 | -2.26 | **0.024** | 0.67 (0.47 ~ 0.95) |  |  |  |  |  |  |

PCR: pathological complete response;POAT: post-operative adjuvant therapy
