# Supplementary material for: Prognostic Value of a Novel Staging System Integrating Lymph Node Station Number and Tumor Regression Grade for Esophageal Cancer Following Neoadjuvant Chemoradiotherapy
Source: Ann Surg Oncol. 2025 Nov 9;33(2):996–1006. doi: 10.1245/s10434-025-18612-y (PMC12765742; doi:10.1245/s10434-025-18612-y)
Supplement: Supplementary file 9 — Supplementary file9 (DOCX 12 kb) [file 10434_2025_18612_MOESM9_ESM.docx]

**Supplementary Table2: NRI between ypTNM and nTRG**

|  | Training Set | |  |  | Test Set | |  |  |
| --- | --- | --- | --- | --- | --- | --- | --- | --- |
|  | Categorical | 95%CI |  | *P*-value | Categorical | 95%CI |  | *P*-value |
| NRI |  |  |  |  |  |  |  |  |
| 12-month | 0.111 | 0.020-0.202 | | 0.017 | 0.257 | 0.070-0.443 | | 0.007 |
| 24-month | 0.111 | 0.019-0.202 | | 0.020 | 0.128 | -0.144-0.399 | | 0.357 |
| 36-month | 0.006 | -0.004-0.057 | | 0.808 | 0.058 | -0.092-0.208 | | 0.445 |

NRI:net reclassification index
